# Development of a prediction score for in-hospital mortality in COVID-19 patients with acute kidney injury: a machine learning approach

**DOI:** 10.1038/s41598-021-03894-5

**Published:** 2021-12-24

**Authors:** Daniela Ponce, Luís Gustavo Modelli de Andrade, Rolando Claure-Del Granado, Alejandro Ferreiro-Fuentes, Raul Lombardi

**Affiliations:** 1 Department of Internal Medicine, Botucatu Medical School, University of São Paulo State–UNESP, Avenida Professor Mario Rubens Montenegro, Botucatu, São Paulo 18618-687 Brazil; 2 Division of Nephrology, Hospital Obrero No. 2 − CNS, Universidad Mayor de San Simon, School of Medicine, Cochabamba, Bolivia; 3 Division of Nephrology, School of Medicine, Universidad de La República, Montevideo, Uruguay

**Keywords:** Kidney diseases, Risk factors

## Abstract

Acute kidney injury (AKI) is frequently associated with COVID-19 and it is considered an indicator of disease severity. This study aimed to develop a prognostic score for predicting in-hospital mortality in COVID-19 patients with AKI (AKI-COV score). This was a cross-sectional multicentre prospective cohort study in the Latin America AKI COVID-19 Registry. A total of 870 COVID-19 patients with AKI defined according to the KDIGO were included between 1 May 2020 and 31 December 2020. We evaluated four categories of predictor variables that were available at the time of the diagnosis of AKI: (1) demographic data; (2) comorbidities and conditions at admission; (3) laboratory exams within 24 h; and (4) characteristics and causes of AKI. We used a machine learning approach to fit models in the training set using tenfold cross-validation and validated the accuracy using the area under the receiver operating characteristic curve (AUC-ROC). The coefficients of the best model (Elastic Net) were used to build the predictive AKI-COV score. The AKI-COV score had an AUC-ROC of 0.823 (95% CI 0.761–0.885) in the validation cohort. The use of the AKI-COV score may assist healthcare workers in identifying hospitalized COVID-19 patients with AKI that may require more intensive monitoring and can be used for resource allocation.

## Introduction

The coronavirus disease 2019 (COVID-19), which is caused by the severe acute respiratory syndrome coronavirus 2 (SARS-CoV-2), has rapidly spread globally. America has been the epicentre of the COVID-19 pandemic for the past few months, and Brazil has the third highest total number of COVID-19 cases worldwide and the second highest number of deaths. The impact of COVID-19 has been devastating on Latin America, with all regions and all states being affected^1,2^. As of 1 March 2021, there are over 20 million confirmed cases and 650,000 deaths, and these figures are probably underestimated^3^.

The estimated incidence of acute kidney injury (AKI) among patients hospitalized for COVID-19 varies between studies, ranging from 0.5% to as high as 60%^4–6^. A Brazilian study was published recently and showed an overall AKI incidence of 50%. Among intensive care unit (ICU) patients, AKI occurred in 77.3% and the mortality was 65.4%^7^.

Many studies have shown that COVID-19 patients who developed AKI had an increased mortality risk^4–8^. A recent study of 5,449 individuals admitted to hospitals across New York found that 35% of patients who developed AKI died, with an adjusted odds ratio (OR) of 9.6^6^.

It is important to provide an accurate estimation of mortality in COVID-19 patients with AKI and to explore the differences in these estimations. This approach will improve treatment strategies and facilitate healthcare planning.

Predictive scores have been developed to assist with risk stratification in patients with AKI or COVID-19^9–12^. Additionally, the predictive models could be particularly useful in supporting the process of decision-making regarding which patient needs urgent assessment.

To date, there is no predictive score for assessing the risk of death in COVID-19 patients with AKI. The present study aimed to develop and validate a prognostic model for AKI associated with COVID-19 patients using data from the Latin America COVID-AKI Registry^13^.

## Patients and methods

This study is part of the Latin America COVID-AKI Registry, an observational, prospective, longitudinal, multinational registry that includes COVID-19 patients with AKI in Latin America^13^. An open invitation to participate was made through the webpage of the Latin American Society of Nephrology and Hypertension (SLANH), the Regional and National Societies of Nephrology and by personal email to members of *RedIRA* (an educational tool of SLANH). Participation in the registry was voluntary, without any incentive or economic benefit for patients or investigators. The Latin America AKI COVID-19 Registry has been conducted in 57 cities in 12 countries from Latin America (60% of the countries in Latin America).

### Bioethical considerations

The Institutional Review Board of the *Clínica Los Olivos*, Cochabamba, Bolivia (contact Dr Esdenka Vega, administracion@clinicalosolivos.com), approved the study. The informed consent was waived due to the observational characteristic of the study by the Review Board of the *Clínica Los Olivos*. The protocol and forms are available on the study’s website (https://slanh.net/registro-latinoamericano-ira-covid-19/). Confidentiality of information was appropriately protected by the de-identification of data. No personal data from the patients were included in the form. All methods were performed in accordance with the relevant guidelines and regulations of the Review Board of the *Clínica Los Olivos*.

Inclusion criteria were patients aged more than 18 years with SARS-CoV-2 infection confirmed by RT-PCR of nasopharyngeal swabs and acute kidney injury (AKI) from 1 May 2020 to 31 December 2020. Exclusion criteria were patients with CKD stage 5, on dialysis or with a transplant.

### Data collection

Data were obtained from the clinical record and were entered online in a SurveyMonkey® platform specifically designed for this purpose (https://es.surveymonkey.com/r/L6PVMGQ).

The data collection sheet had the following variables: (1) country and city of residence, demographic data; (2) comorbidities and condition at admission; (3) laboratory values at admission; (4) characteristics and aetiology of AKI; (5) ICU admission, mechanical ventilation (MV) and in-hospital complications; and (6) condition at discharge.

### Definitions

AKI was identified according to the KDIGO and defined by an increase in serum creatinine (SCr) level ≥ 0.3 mg/dl within 48 h or by 50% within 7 days. AKI was considered community-acquired (CA-AKI) when it was present at admission or within 48 h of hospital admission. AKI was considered hospital-acquired (HA-AKI) when it developed greater than 48 h into the hospital stay^14^.

In cases with no pre-admission Scr level, we assumed its value at admission or within the first 24 h in a patient without chronic kidney disease (CKD)^15,16^.

Condition at admission to the hospital was classified into three categories: mild, if the patient was admitted to a conventional ward without need for oxygen therapy; moderate, if the patient needed oxygen therapy; and severe, when admission to the intensive care unit (ICU) was required.

The study followed the guidelines of the transparent reporting of a multivariable prediction model for individual prognosis or diagnosis (TRIPOD)^17^.

### Predictor variables

We evaluated the predictors available at the time of COVID-19 diagnosis that were related to four categories: (1) demographic data; (2) comorbidities and condition at admission; (3) laboratory examinations within 24 h; and (4) aetiology of AKI (Supplementary Material Table 1). The laboratory examinations and haemodynamic parameters were collected within 24 h of admission. The comorbidities evaluated were diabetes, obesity, hypertension, lung disease, neoplasia, liver disease, autoimmune disease, cardiovascular disease, and smoking. We also evaluated the time from COVID-19 symptoms to hospitalization and the time from hospitalization to AKI. The aetiology of AKI was determined by the attending nephrologists using a diagnostic workup that included medical history, clinical examination, renal ultrasound, urinalysis, and blood testing to either classify the aetiology of AKI as ischaemic, septic or nephrotoxic (and therefore not associated with SARS-CoV-2 infection).

### Outcome

The outcome evaluated was in-hospital death. Use of medications to treat COVID-19, admission to the ICU, and need for MV were assessed at each centre according to their own local practices.

### Statistical analysis

#### Exploratory data analysis

All variables of interest were compared between patients who survived and those who died in the hospital. This comparison was performed by the χ^2^ test for categorical variables and by the Mann–Whitney test for continuous variables.

#### Predictive model

For the predictive model, the categorical variables were transformed into dummy variables. We removed the variables with more than 30% missing values (18% of the predictors) and imputed the others (Supplementary Fig. 1 and Table 1). We used a k-nearest neighbours (KNN) algorithm for the imputation method to account for missing values. We used all predictor variables to compute the Gower's distance and five nearest neighbours in the KNN imputation model. Once the nearest neighbours were determined, the mode was used to impute nominal variables, and the mean was used for numeric data.

The continuous variables were normalised by dividing their values by the mean (centre) and standard deviation (scale). We transformed continuous variables using Box-Cox transformations. Variables with zero or near-zero variance were removed from the model.

#### Feature selection

We used the Boruta algorithm to select the most important predictors. The Boruta algorithm is a feature selection method that classifies which of the features are important and which are not. The algorithm uses feature importance scores, which are provided by random forest. The importance measure of an attribute is obtained as the loss of accuracy of classification caused by the random permutation of attribute values between objects. It is computed separately for all trees in the forest that use a given attribute for classification. Then, the average and standard deviation of the accuracy loss are computed. The method performs a top-down search for relevant features by comparing the importance of original attributes and progressively eliminating irrelevant features^18^. We removed the features considered not important by the Boruta algorithm and highly correlated the variables (correlation value above 0.9) (Supplementary Table 1).

#### Model training

We split the data into derivation (training) and validation (test) datasets. To create the datasets, we used a random split stratified by the target into training (80%) and test (20%). In the training data (derivation cohort), tenfold cross-validation was used to select the hyperparameters of the models and to reduce the bias. The data were randomly partitioned into 10 sets of roughly equal size. For each iteration, one fold was held out for assessment statistics and the remaining folds were substrate for the model. This process continued for each fold so that the models produced 10 sets of performance statistics.

We fitted gradient boosting decision trees (XGBoost), random forest, and an Elastic Net to develop the candidate equations. Finally, the best hyperparameters were selected using machine learning approaches by tenfold cross-validation in a train set aimed to maximize the area under the receiver operating characteristic curve (AUC-ROC).

#### Assessment of accuracy

The accuracy of the derivation cohort model was tested on the data of the validation cohort. We used the AUC-ROC to discriminate the ability of the models in the train and test sets. The 95% confidence interval (CI) of the AUC-ROC was estimated by bootstrap resampling (2,000 samples) to reduce over-fit bias. We evaluated the calibration of models with the Brier score and a calibration plot (Supplementary Fig. 2).

### Accuracy metrics for previously published models

The final model was compared with two available models that have been externally validated in the general population: the CHA2DS2-VASc^19^ and the clinical predictive model proposed by Wang et al.^20^. The CHA2DS2-VASc is used to estimate thromboembolic stroke risk in atrial fibrillation and thrombotic risk in cardiac diseases and was evaluated in patients diagnosed with COVID-19. The second cited model was derived from a cohort of patients diagnosed between January 2020 and February 2020, in Wuhan, and was built by the XGBoost approach, considering age, history of hypertension, and history of coronary disease as predictors. Finally, we compared the performance of the final model with the Acute Tubular Necrosis Index Specific Score (ATN-ISS). The ATN-ISS^21^ is a validated general score to predict mortality in patients with AKI. The comparisons were performed throughout the assessment of sensitivity, specificity, and AUC-ROC.

### Sensitivity analysis

For the sensitivity analysis, we fitted the LASSO algorithm using all available predictors (without using feature selection). We also fitted the LASSO with the predictors that had more than 30% missing data. The LASSO algorithm automatically selects those features that are useful, discarding the useless or redundant features. The analysis was performed by the AUC-ROC.

#### Score fit and model visualization

The model with higher AUC-ROC in the validation cohort associated with a better calibration value was used to build the AKI-COV score. We used Shapley Additive Explanations (SHAP) to visualise and explain the importance of predictors.

The software R version 4.0.2 and the packages tidymodels and DALEx were used to create and visualise the models. The R package “glmnet” statistical software (R Foundation) was used to perform the Elastic Net regression^22^.

The R programming code and the final model are available in a supplementary file. The steps to validate the model in an external cohort are described in the supplementary materials.

### Ethical approval

Not applicable (register study).

### Consent to participate

Not applicable (register study).

### Consent for publication

All authors declare to approve the paper for publication.

### Transparency declaration

The lead authors (DP and RL) confirm that the manuscript is an honest, accurate, and transparent account of the study being reported; that no important aspects of the study have been omitted; and that any discrepancies from the study as originally planned (and, if relevant, registered) have been explained.

## Results

### Demographic data, COVID-19 infection presentation and difference between survivor and non-survivor AKI patients

Between May 2020 and December 2020, 967 COVID-19 patients with AKI were identified in 57 centres and 12 countries from Latin America that were part of the SLANH COVID-19-AKI Registry. Of 967 patients, we retrieved and analysed 870 patients with outcome mortality data available.

The median age of patients was 63 (54–74) years, 595 patients were male (68.4%), and 759 (87.2%) patients had one or more comorbidities, with hypertension, diabetes and obesity being prevalent (Table 1). The time between diagnosis of COVID-19 and hospital admission was 2 (0–4) days and the condition at hospital admission was mild in 121 (14.0%) patients, moderate in 384 (44.2%) patients and severe in 363 (41.8%) patients. Of note, about half of patients had a serum creatinine (sCr) at admission within normal values.

**Table 1:** Demographic and clinical characteristics of COVID-19 patients with Acute Kidney Injury at hospital admission.

Variable	General N = 870	Non-survival N = 544	Survival N = 326	*p*
Male sex (%)	595 (69)	383 (71)	212 (65)	0.1
Age	63 2 ± 14.8	65.1 ± 13.8	59.2 ± 16.1	0.0001
**Comorbidities (%)**				
Cardiovascular disease	135(15.5)	94 (17.3)	41 (12.6)	0.038
Obesity	278 (31.9)	199 (36.6)	79 (24.2)	0.0001
**Condition at admission n (%)**				
Mild	121 (13.9)	36 (6.6)	85 (26.2)	0.0001
Moderate	384 (44.1)	237 (43.6)	147 (45.2)	
Severe	363 (41.7)	270 (49.7)	93 (28.6)	
**Laboratory exams at hospital admission**				
White blood cell count (mm^3^)	11,807 ± 5518	12,607 ± 6578	10,439 ± 2585	0.0001
Lymphocytes/mm^3^	862 ± 258	850 ± 201	871 ± 287	0.02
Ferritine (ng/ml)	1285 ± 1021	1435 ± 1126	1055 ± 1022	0.0001
CK (U/L)	411 (289–534)	744 (576 -1433)	414(227–602)	0.004
D-Dimer (ng/mL)	1,132 ± 310	1,298 ± 337	1,030 ± 202	0.5
Time from COVID symptoms to hospitalization (days)	4.0 (1.0–6.0)	2 (0.0–3.0)	5.0 (1.0–6.0)	0.18
Hospital-acquired AKI n (%)	547 (62.8)	399 (74.0)	148 (48.2)	0.0001
**Cause of AKI n (%)**				
Hypovolemia	311 (35.7)	156 (28.7)	155 (47.59	0.0001
MODS SARS-CoV2	515 (59.2)	376 (69.1)	139 (42.6)	0.0001
MODS sepsis	254 (29.2)	207 (38.1)	47 (14.4)	0.0001
Nephrotoxic drugs	182 (20.9)	99 (18.2)	83 (25.5)	0.007
Oliguric AKI (%)	336 (38.6)	259 (48.3)	77 (25.0)	0.0001
Scr peak mg/dL	3.88 (2.5–5.1)	4.30 (2.9–6.3)	3.17 (2.1–4.1)	0.0001
Kidney replacement therapy (%)	402 (46.2)	315 (58.0)	87 (27.0)	0.0001
Non-recovery of renal function	533 (61.3)	462 (88.5)	71 (23.7)	0.0001
Duration of kidney replacement therapy (days)	12.4 (6.89–16.90)	8.19 (4.98—10.12)	16.5 (11.7–21.20	0.0001
Admission to ICU (%)	622 (71.5)	484 (88.9)	138 (42.3)	0.0001
Mechanical ventilation (%)	628 (72.2)	492 (90.6)	136 (42.1)	0.0001
Use of vasopressors (%)	527 (60)	432 (82)	95 (29)	0.0001
Minimum PaO_2_/FiO_2_	159 ± 66	140 ± 46	190 ± 82	0.0001
New onset of proteinuria	163 (18.7)	106 (32.8)	57 (24.1)	0.015
New onset of hematuria	133(15.3)	101 (30.9)	32 (13.7)	0.0001
Last available Scr mg/dL	3.18(1.54–3.83)	3.74(1.74–4.02)	2.17(1.31–2.90)	0.0001
**Complications (%)**				
Sepsis	439 (50.4)	360 (67.2)	79 (25.1)	0.0001
Infection	76 (8.73)	22 (4.3)	54 (17.4)	0.0001
other complications	161 (18.5)	51 (9.9)	110 (34.9)	0.0001
Hospital stay (days)	19.6 (13.8–23.9)	16.2 (12.6–18.5)	21.6 (15.5–26.1)	0.0001
Time from COVID symptoms to AKI (days)	5.0 (1.0–8.0)	4 (1.0–6.0)	7.0 (0.0–3.5)	0.18

CK: creatinophosphokinase; AKI: acute kidney injury; ICU: intensive care unit; MODS: multiple organs disfunction syndrome; Scr: serum creatinine.

AKI was hospital-acquired in 547 (62.9%) patients. The time between diagnosis of COVID-19 and the onset of AKI was 3 (1–7) days. Multiple organ dysfunction syndrome (MODS) attributable to SARS-CoV-2 infection was the main cause of AKI (35.7%). As expected, in many patients, AKI was linked to more than one aetiology. In the majority of cases, AKI was non-oliguric (59.9%). Most patients had AKI-KDIGO 3 (59.7%), followed by AKI-KDIGO 1 (25.8%). Kidney replacement therapy (KRT) was performed in 402 patients (46.2%). The most common KRT was intermittent haemodialysis (IHD) (69.1%), followed by prolonged intermittent renal replacement therapy (PIRRT) (32.8%), continuous renal replacement therapy (CRRT) (15.6%) and peritoneal dialysis (PD) (2.7%). Patients were treated for SARS-CoV-2 infection with steroids (73.9%), chloroquine-hydroxychloroquine (12.5%), ivermectin (6.8%), tocilizumab (1.5%), and remdesivir and lopinavir-ritonavir (0.6%). It should be highlighted that, in a large proportion of patients, the option “other treatment” was selected in 46.9% of them, with no further specification. Most of the patients had complications during the hospitalization, with sepsis being the most common (50.4%), followed by infection without sepsis (8.7%) and deep vein thrombosis (5.4%).

All-cause in-hospital mortality was 62.5% (544 out of 870 patients). Most of the deaths occurred in the ICU (88.9%). Table 1 shows the variables associated with mortality in the univariate analysis.

### Development of model prediction risk for COVID-19-associated mortality

**Figure 1:**
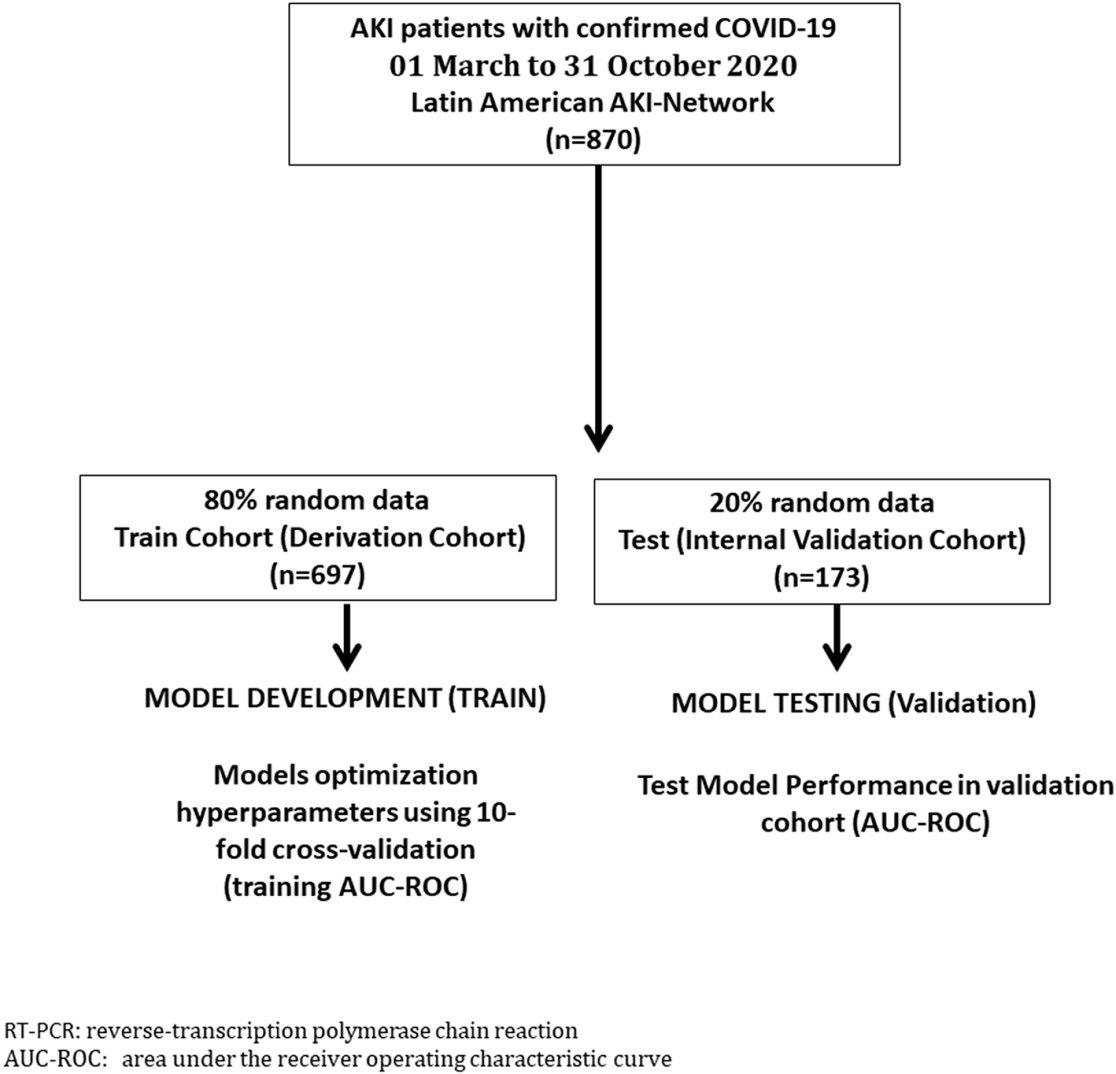
Derivation cohort or train set and the internal validation cohort or test set.

The patients were grouped randomly into two cohorts: the derivation cohort (or train set) (n = 697, 80%) and the internal validation cohort (or test set) (n = 173, 20%), as shown in Fig. 1.

We had a total of 44 predictors and removed seven of them due to higher missing values (> 30%). We also removed 17 uninformative and two collinear predictors (Supplementary Table 1). Then, we fitted predictive models using the candidate predictors (n = 18).

We fitted several candidate models with tenfold cross-validation and analysed the performance of these models throughout the AUC-ROC in the derivation cohort. The AUC-ROC were 0.894 (0.82–0.93), 0.886 (0.85–0.95), and 0.877 (0.83–0.93) for random forest, XGBoost and Elastic Net, respectively. In a second step, the performances of these models were tested in the internal validation cohort. The AUC-ROC was 0.831 (0.76–0.89), 0.823 (0.75–0.88), and 0.821 (0.75–0.88) for random forest, XGBoost and Elastic Net, respectively (Table 2).

**Figure 2:**
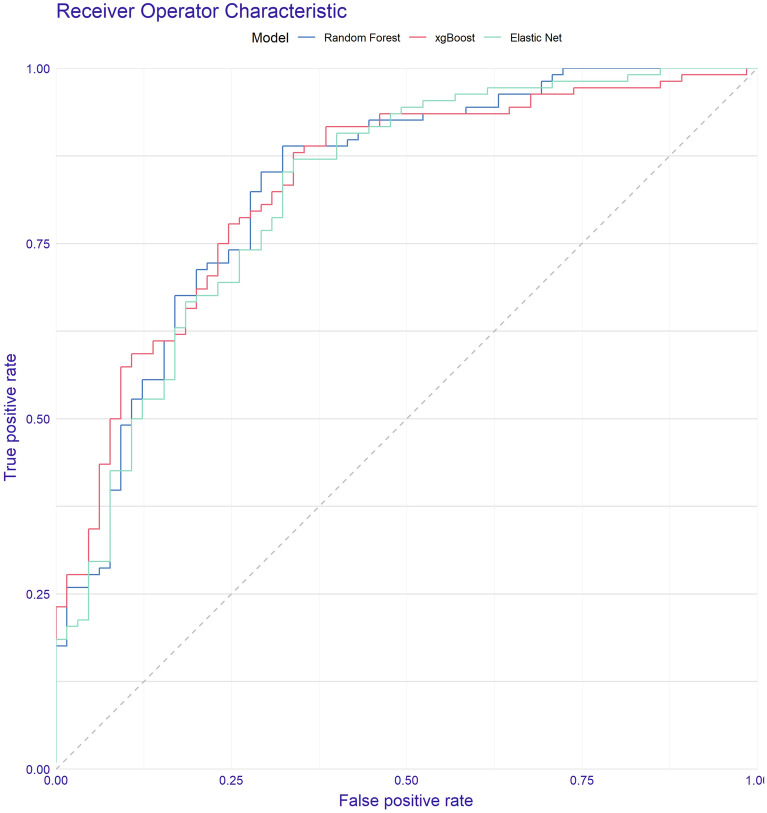
AUC-ROC in the derivation cohort of AKI-COVID-19 mortality predictive models.

**Figure 3:**
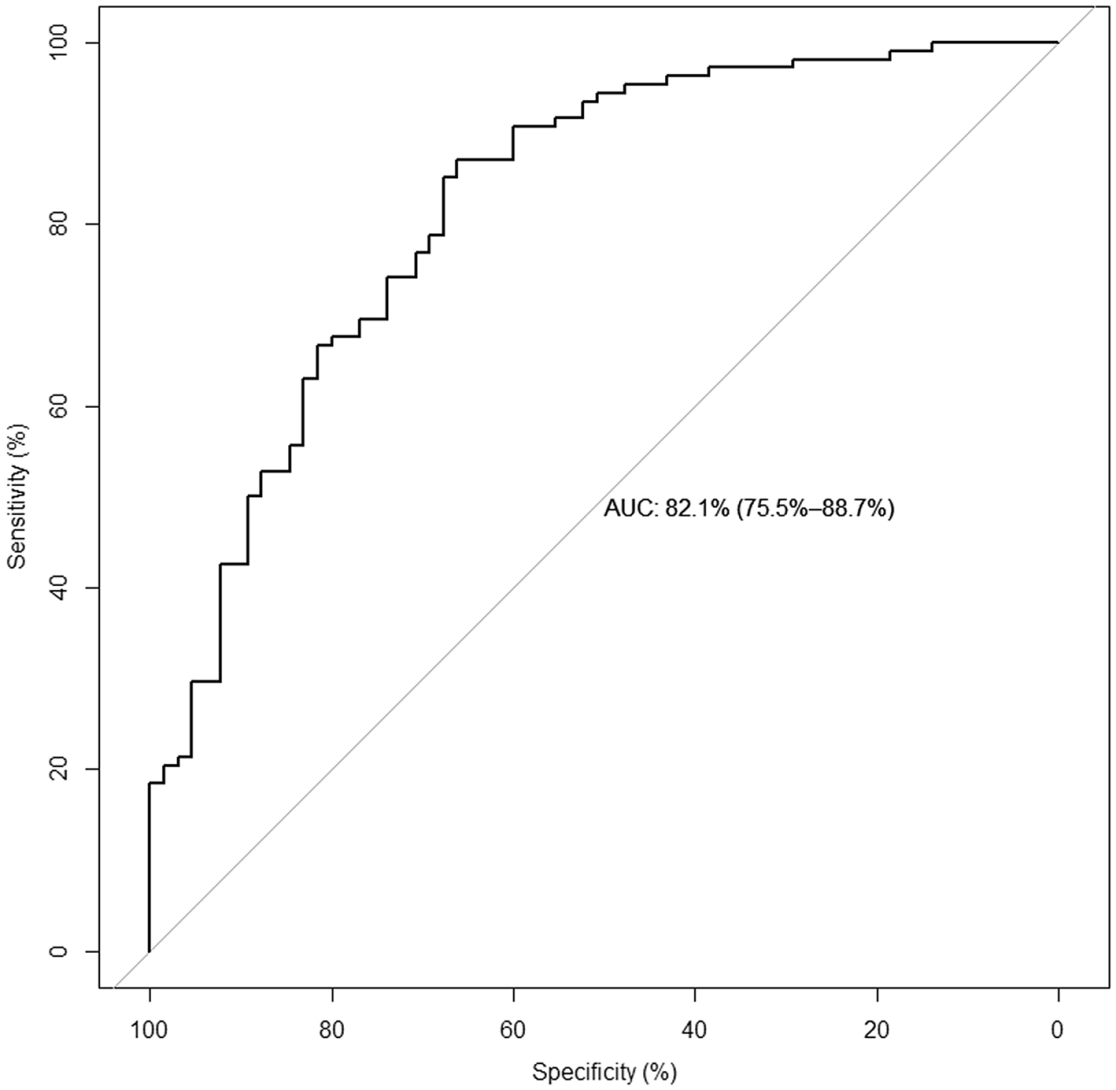
Confusion Matrix of AKI-COVID-19 in hospital mortality in derivation cohort (test set).

To choose the most useful model, we additionally plot the AUC-ROC values in Fig. 2, and a confusion matrix of 28-day mortality in the derivative cohort is shown in Fig. 3. The performance was similar, but the Elastic Net had better calibration values in the validation cohort and was selected as a final model (Supplementary Fig. 2).

The final model fitted (Elastic Net) was a combination of the least absolute shrinkage and selection operator (LASSO) with ridge regression. It was a generalised linear model via penalised maximum likelihood that performs regularisation and variable selection^23^. The regularisation path is computed for the LASSO penalty at a grid of values for the regularisation parameter lambda^23^. The proportion of LASSO and ridge regression, as well as the amount of regularisation, was selected by tenfold cross-validation.

### Making a score-based prediction

**Figure 4:**
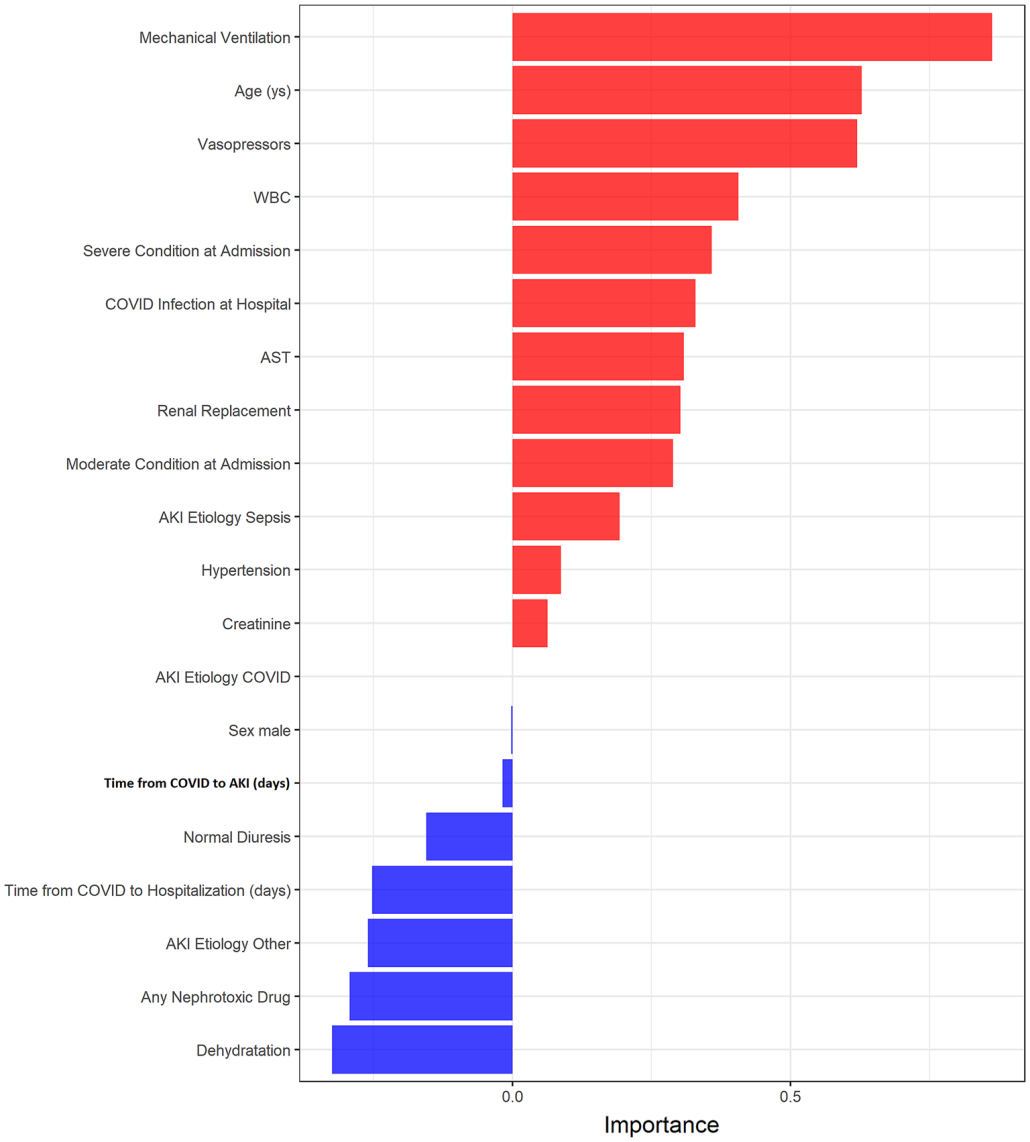
Coefficients of Elastic Net of AKI-COVID-19 in-hospital mortality model (Variable Importance). The red bars represent the variables related with the probability of death, whereas the blue bars were related with the probability of surviving. The model was fitted with 15 predictors and we derived natural splines in the variables age and eGFR. The natural splines computed a different risk for each stratum aiming to capture the non-linear association between these predictors and outcome.

The results of the Elastic Net model showed that MV, higher age, vasopressors, higher leukocyte values, higher aspartate aminotransferase (AST) values, severe/moderate condition at admission, hypertension, HA-AKI, AKI aetiology related to sepsis or COVID-19, higher creatinine levels during hospitalisation and indications for KRT were related to a worse outcome. Higher urine output, longer time from COVID symptoms to hospitalisation, use of nephrotoxic drugs, dehydration and AKI of nephrotoxic or ischaemic aetiology were related to a better outcome (Fig. 4). The coefficients of the Elastic Net model were used to build the AKI-COV score.

### Comparison of accuracy metrics with previous models

The performance of the AKI-COV score was compared with two COVID-19 models derived from the general population and one model used in patients with AKI. The results are shown in Supplementary Table 2. The sensitivity, specificity and AUC-ROC were 0.19, 0.90, and 0.60, respectively, for the CHA2DS2-VASc score; 0.55, 0.56 and 0.57, respectively, for the model derived from the Wuhan cohort; and 0.57, 0.91 and 0.61, respectively, for LIANO score. Therefore, all of them resulted in low specificity and lower AUC-ROC values for patients with AKI, underperforming on the AKI-COV score.

**Table 2:** Performance metrics (AUC-ROC) of COVID-19 mortality models in derivation cohort and validation cohorts.

Model	AUC-ROC	
	Derivation cohort (n = 697)	Internal Validation cohort (n = 173)
**Random Forest**	0.894 [0.82–0.93]	0.831 [0.76–0.89]
**XgBoost**	0.886 [0.85–0.96]	0.823 [0.75–0.88]
**Elastic Net**	0.877 [0.83–0.97]	0.821 [0.75–0.88]

[95% Confidence Interval] based on 2000 bootstrap resample.

### Sensitivity analysis

We performed the sensitivity analysis fitting a LASSO model with all available predictors (n = 44). The sensitivity, specificity and AUC-ROC were 0.73, 0.77, and 0.81, respectively, for LASSO without prior feature selection but excluding higher missing values (n = 36); 0.77, 0.76, and 0.81 for LASSO including the predictors with more than 30% missing values (n = 44) (Supplementary Table 3). These results were similar to the final AKI-COV model. Then, we chose AKI-COV as the final model because of similar performance using fewer predictors.

### Practical application

**Figure 5:**
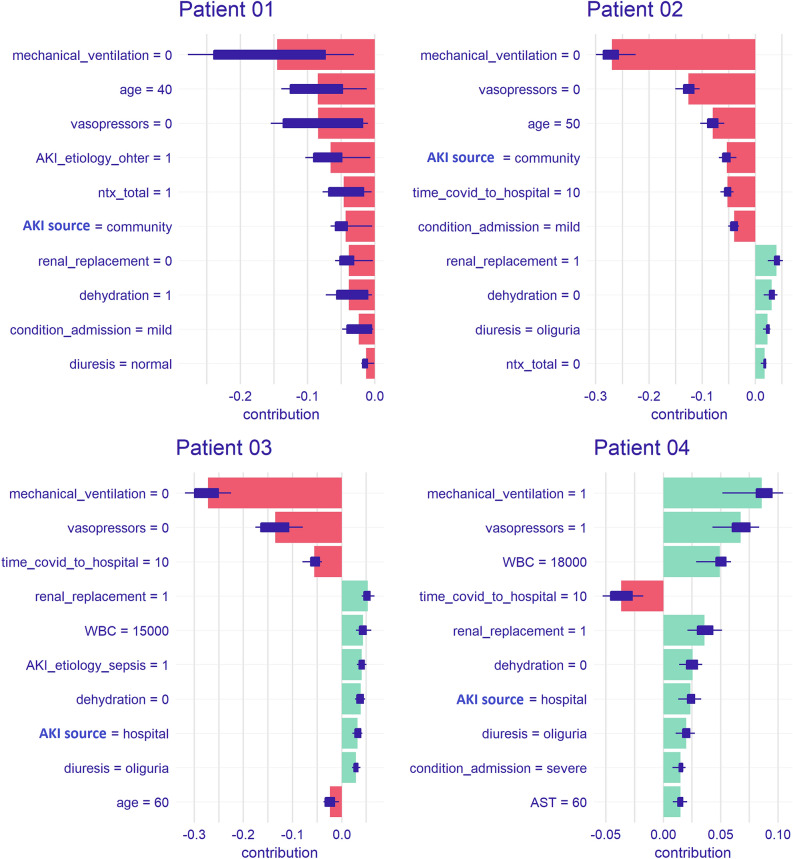
Shapley Additive Explanations (SHAP plot) showed the contribution of each predictor in AKI COVID-19 in-hospital mortality score.

We show examples of predictions in four different hypothetical patients (Table 3). Patients 1 and 2 had the same CA-AKI and mild condition at admission, while patients 3 and 4 had HA-AKI and a moderate/severe condition at admission. There was KRT in patients 2 to 4 and MV and vasopressors in patient 4. In these scenarios, the probability of death was progressive, ranging from 0.5% to 93%. For better demonstration, the contribution and importance of each predictor are visualized in a SHAP plot, as shown in Fig. 5.

**Table 3:** COVID-19 mortality prediction (AKI-COV) in four hypothetical AKI patient.

	Patient 1	Patient 2	Patient 3	Patient 4
**DEMOGRAPHY**				
Age (yeas)	40	50	60	60
Sex male	Yes	Yes	Yes	Ye
Hypertension	No	No	No	No
**Time from COVID symptoms to:**				
Hospital admission (days)	5	10	10	10
AKI (days)	10	12	12	12
Acquired AKI in	Community	Community	Hospital	Hospital
Condition at admission	Mild	Mild	Moderate	Severe
Dehydratation	Yes	No	No	No
Use of Nefrotoxic Drugs	Yes	No	No	No
AKI etiology	Other	COVID	Sepsis	COVID
Diuresis	Normal	Oliguria	Oliguria	Oliguria
**Laboratory**				
WBC	10,000	12,000	15,000	18,000
AST	40	50	60	60
Creatinine	2	2.5	3	2.5
Indication of Renal Replacement	No	Yes	Yes	Yes
Mechanical Ventilation	No	No	No	Yes
Use of Vasopressors	No	No	No	Yes
**PREDICTIONS**				
Probability 28 days Death	**0.5%**	**12.7%**	**40.8%**	**93%**

AKI: acute kidney injury; AST: aspartate aminotransferase; WBC: white blood cell counts.

Finally, we developed a web app to estimate the individual probability for point-of-care decisions, which is available at: https://covidmodels.shinyapps.io/covid_aki_app/.

## Discussion

Previous studies have used machine learning to develop COVID-19 prognostic models with overall good performance^24,25^, frequently reaching over 0.90 AUC-ROC^26^. However, to the best of our knowledge, this is the first multicentre study that developed a model named AKI-COV to predict in-hospital COVID-19 mortality in patients with AKI. The model was performed and validated in a cohort in Latin America, the new epicentre of the COVID-19 pandemic. In this model, we used predictors that are easily available upon initial diagnosis and at hospital admission. The AKI-COV score achieved a higher discriminative capacity to predict patient mortality and can contribute to the early start of interventions, thereby increasing the survival of COVID-19 with AKI^23,26,27^.

Using variable importance analysis, we identified the predictors associated with mortality in the COVID-19 patients with AKI. Higher age, need for MV, use of vasopressors, higher leukocyte values, higher ALT values, severe/moderate condition at admission, hypertension, HA-AKI, AKI aetiology related to sepsis or COVID-19, and an indication of KRT were related to a worse outcome. On the contrary, higher urine output, shorter time from COVID-19 symptoms to hospitalization and AKI diagnosis, use of nephrotoxic drugs, dehydration, and nephrotoxic aetiology of AKI were related to a better outcome (Fig. 4).

Higher age and leukocytosis were associated with negative outcomes in critically ill patients^28,29^ and were also associated with poor outcomes in SARS-CoV-2 patients^30,31^. Aging is associated with a well-known decrease in immunity, which leads to an increased susceptibility to infections^28^. Additionally, age-related immune imbalance may be present, leading to increased production of cytokines, which enhances the vulnerability of patients as a result of the cytokine storm in response to COVID-19^30^.

Classical comorbidities (such as hypertension), need for MV, and use of vasopressors are predictors already associated with higher mortality in patients with AKI or COVID-19^32,33^.

The presence of comorbidities, such as diabetes and hypertension, indicate the importance of the effects of pre-existing conditions on the severity of COVID-19 and AKI. Even though hypertension is age-dependent, it remained an independent risk factor in the final AKI-COV model.

We showed that hospital-acquired (HA-AKI) was related to a worse outcome, similar to the reports of non-COVID-19 patients^34^. This may be related to higher frequencies of both the severe condition and admissions to the ICU, which may lead to an increase in sepsis-associated AKI. Additionally, these patients present with higher comorbidities and increased age^35^.

We showed that both the longer time from COVID-19 symptoms to hospitalization and time from COVID-19 to AKI were associated with a better outcome. Similar results were observed by others, suggesting that the longer time from COVID-19 symptoms to the presentation may reflect a less aggressive disease^36^.

The AKI-COV model retrieved an AUC-ROC of 0.82, which was classified as a good performance, considering the reduced number of predictors. Other predictive models of COVID-19 retrieved a better accuracy but combined data from vital signs, radiology exams, and laboratory exams, which can also limit its application. A review of COVID-19 predictive models concluded that the majority were of low quality. The reasons can be summarized in the absence of a description of the study population, absence of calibration plots, and absence of a validation cohort^37^. In contrast, we used a machine learning approach combined with a well-described cohort of patients.

### Possible applications

Using predictors available at baseline and within the first hours of the admission, we could objectively predict the probability of death of a COVID-19 patient with AKI. With an accurate prediction, we could allocate resources to patients who we expect to have a better prognosis^38^. In the scenario of the COVID-19 pandemic, the critical decision to not perform dialysis because of lack of resources was made in several countries. Of note, in the present cohort, some patients needing KRT did not receive it. There were 43 people identified meeting this condition (4.9%). Reasons were not recorded but were presumably due to resource shortages.

On the opposite, another application was a change in a worse outcome using the scores of AKI-COV. For example, a patient presenting with a higher score in AKI-COV had a higher rate of death. We could anticipate dialysis or propose a more advanced resource like continuous renal replacement therapy aimed to reduce this unfavored prognostic. These interventions could possibly modify the unfavored outcome if the resources were available.

A major strength of the AKI-COV score is its simplicity; the use of objective parameters, which may reduce the variability; and easy availability, even in under-resourced settings. Then, the AKI-COV score may help clinicians to make a prompt and reasonable decision to optimize the management of COVID-19 patients with AKI and potentially reduce mortality.

## Limitations

Our study has a few limitations. First, we analysed data from Latin America at different time points. In these periods, the clinical protocols for COVID-19 were still being established, so this could affect the outcomes and caution should be used when generalizing results. Obesity was not directly measured by body mass index, but was rather clinically defined, as its measurement was based on medical records, which may have led to underreporting. Due to the pragmatic study design, laboratory exams were performed according to physician order, which can contribute to higher proportions of missing data. Additionally, some laboratory parameters, which proved to be of prognostic relevance in other studies, were not evaluated. Additionally, the time from COVID-19 symptoms to hospitalization was not directly measured but retrieved from questionnaires applied to patients or their families. Another limitation was that the diagnosis of AKI was based on clinical presentation, rather than biopsy proven.

Moreover, the machine learning models had a primary focus in prediction with lower explanatory capacity compared to classic statistical analysis. This could reduce the inferential conclusions; therefore, additional studies are required to determine the impact of the associated predictors of mortality in COVID-19 patients with AKI. For these reasons, study designs that reduce causal inference may be useful^39–42^.

## Conclusion

In conclusion, we developed and validated a model named AKI-COV to predict the in-hospital mortality of COVID-19 patients with AKI. This score used a few predictors available at baseline that retrieved a good accuracy. This could be an inexpensive tool to predict the in-hospital mortality of COVID-19 patients with AKI objectively and accurately. Additionally, it may be used to inform clinical decisions and the assignment to the appropriate level of care and treatment for COVID-19 patients with AKI.

### Latin American AKI COVID-19 Registry investigators (in alphabetical order)

Adriana Flores (Mexico City, Mexico), Alejandro Ferreiro-Fuentes (Montevideo, Uruguay), Ana Paula Villa (Guadalajara, Mexico), Aureliano Ferrari (Asuncion, Paraguay), Benedito Pereira (Sao Paulo, Brazil), Caio Costa (Petropolis, Brazil), Carlos F. Varela (Buenos Aires, Argentina), Caroline de Acevedo (Rio de Janeiro, Brazil), Cristina Carlino (Rosario, Argentina), Daniela Ponce (Botucatu, Brazil), Diego Janiques (Petrópolis, Brazil), Emmanuel Burdmann (Sao Paulo,Brazil), Eric Roessler (Santiago de Chile, Chile), Giannyigg (ICA, Peru), Giovanni Arrivillaga (Quetzaltenango, Guatemala), Gisselle Guzman (Santo Domingo, Dominican Republic), Galo Andrés Coronel (Buenos Aires, Argentina), Guillermo Rosa-Diez (Buenos Aires, Argentina), Gustavo Aroca (Barranquilla, Colombia), Jonathan Chavez (Guadalajara, Mexico), Jose Ubillo (Mexico City, Mexico), Julieta Raño (Buenos Aires, Argentina), Lilia Rizo-Topete (Monterrey, Mexico), Luis Rodríguez (Tucumán, Argentina), Luis Yu (Sao Paulo, Brasil), Marcos Colombo (Jau, Brazil), Mariana B. Pereira (Sao Paulo, Brazil), Mauricio Younes-Ibrahim (Rio de Janeiro, Brazil), Natalia Rivas (Rojas, Argentina), Nelson Rojas (Buenos Aires, Argentina), Roger Ayala (Asuncion, Paraguay), Raúl Ivan Nava (Bolivia), Raúl Lombardi (Montevideo, Uruguay), Rolando Claure-Del Granado (Cochabamba, Bolivia), Serena Amor (Montevideo, Uruguay), Washington Osorio (Quito, Ecuador), Yanissa Venegas (Lima, Peru).

## Supplementary Information

Supplementary Information 1.

Supplementary Information 2.

Supplementary Information 3.

Supplementary Information 4.

Supplementary Information 5.

## Data Availability

Data are available upon reasonable request.
