# Supplementary material for: Development of a prediction score for in-hospital mortality in COVID-19 patients with acute kidney injury: a machine learning approach
Source: Sci Rep. 2021 Dec 24;11:24439. doi: 10.1038/s41598-021-03894-5 (PMC8709848; doi:10.1038/s41598-021-03894-5)
Supplement: Supplementary file 4 — Supplementary Information 4. [file 41598_2021_3894_MOESM4_ESM.docx]

## Missing Data Information

**Figure 01. Plot of missing data. The back color represents the missing data.**


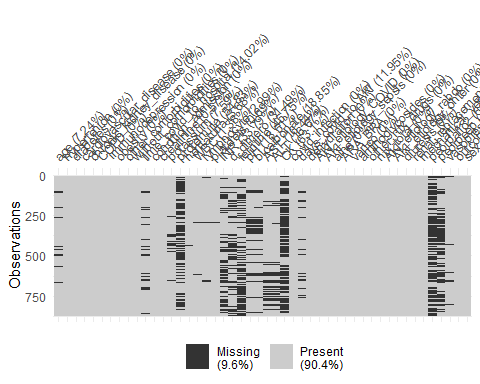


**Table 01**. **Available predictors, percentage of missing data and decision to remove of the model fit**

| Variable | Class | Decision | % Missing |
| --- | --- | --- | --- |
| Age | predictor | Confirmed | 7.2 |
| Sex male | predictor | Confirmed | 0.5 |
| Hypertension | predictor | Confirmed | 0 |
| Diabetes | predictor | Removed* | 0 |
| Cardiovascular Disease | predictor | Removed* | 0 |
| Chronic Kidney Disease | predictor | Removed* | 0 |
| COPD | predictor | Removed* | 0 |
| Immunodepression | predictor | Removed* | 0 |
| Obesity | predictor | Removed* | 0 |
| Other comorbidities | predictor | Removed* | 0 |
| Without comorbidities | predictor | Removed* | 0 |
| Time from COVID to Hospital (days) | predictor | Confirmed | 14 |
| Time from COVID to AKI (days) | predictor | Confirmed | 12 |
| Creatinine | predictor | Confirmed | 0.6 |
| Potassium | predictor | Removed& | 7.6 |
| Hematuria | predictor | Removed* | 54.8 |
| WBC | predictor | Confirmed | 1.4 |
| Lymphocyte | predictor | Removed& | 3 |
| Platelets | predictor | Removed* | 0.7 |
| CK | predictor | Removed+ | 63.1 |
| Ferritin | predictor | Removed+ | 45.7 |
| INR | predictor | Removed+ | 33.8 |
| D dimer | predictor | Removed+ | 31.5 |
| proteinuria | predictor | Removed+ | 54.8 |
| pH | predictor | Removed* | 18.5 |
| Bicarbonate | predictor | Removed* | 18.9 |
| ASL | predictor | Confirmed | 16.4 |
| ALT | predictor | Removed& | 16.7 |
| AKI etiology | predictor | Confirmed | 0 |
| Use ARA or BRA | predictor | Removed* | 0 |
| Use AINES | predictor | Removed* | 0 |
| Use aminoglycosides | predictor | Removed* | 0 |
| Use Vancomycin | predictor | Removed* | 0 |
| Use another nephrotoxic drug | predictor | Removed* | 0 |
| Use any nephrotoxic drug | predictor | Confirmed | 0 |
| Condition at admission | predictor | Confirmed | 0 |
| Local COVID infection | predictor | Confirmed | 0 |
| Dehydration | predictor | Confirmed | 0 |
| Diuresis | predictor | Confirmed | 0 |
| Indication of renal replacement | predictor | Confirmed | 0 |
| Use of vasopressors | predictor | Confirmed | 0 |
| Mechanical Ventilation | predictor | Confirmed | 0 |
| peorpafio2 | predictor | Removed+ | 61.4 |
| peorpeep | predictor | Removed+ | 45.1 |
| TOTAL |  | 44 | 9.6 |
| Removed unimportant* |  | 17 |  |
| Removed collinearity& |  | 2 |  |
| Removed missing > 30%+ |  | 7 |  |
| Total analyzed |  | 18 |  |

* unimportant predictors (Boruta Algorithm)

& collinearity predictors

+ more than 30% missing values

**Figure 2A. Calibration Plot of Random Forest Model**


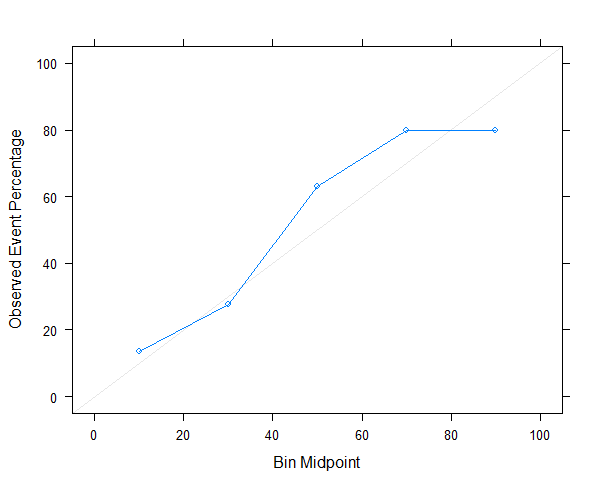


**Figure 2B. Calibration Plot of xgBoost Model**


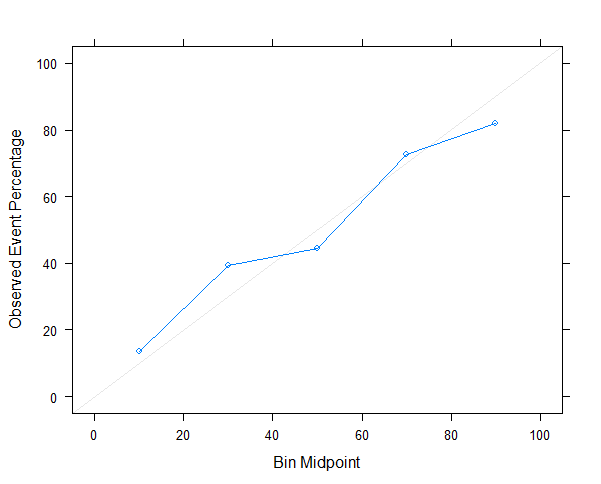


**Figure 2C. Calibration Plot of Elastic Net Model**
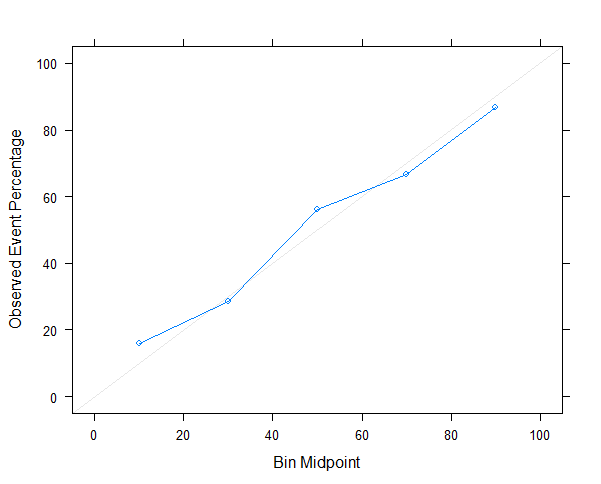


**Supplementary Table 02. Comparison of accuracy metrics with previous predictive model**

| Model | Population | External validation | Parameters IN THE MODEL | Sensitivity | Specificity | Positive  predictive value | Negative  predictive value | ROC AUC* |
| --- | --- | --- | --- | --- | --- | --- | --- | --- |
| CHA2DS2-VASc score | COVID-19 patients (n=1000) | The authors used a previous validated model in a COVID-19 population | 1.Age  2.Hypertension  3.Diabetes  4.Previous Stroke or transient ischemic attack | 0.19 | 0.90 | 0.56 | 0.65 | 0.60 |
| Clinical Model | A Cohort Study in Wuhan, China (n=296) | Validation cohort (n=44) | 1.Age  2.History of hypertension  3.History of coronary disease | 0.55 | 0.56 | 0.43 | 0.67 | 0.57 |
| LIANO | 228 AKI patients | Derivation cohort (n=228)  Validation cohort (n=100) | 1.Age  2.Sex  4.Nephrotoxicity  5.Oliguria  6.Hypotension  7. Jaundice  8. Coma  9. Assisted Respiration | 0.57 | 0.91 | 0.80 | 0.78 | 0.61 |
| AKI-COV SCORE  (Current Model) | COVID-19 AKI patients in Latin America (n=870) |  | 1. Age  2. Condition at Admission  3. AKI etiology  4. Hypertension  5. Sex  6. AKI source  7. Time COVID to AKI  8. Time COVID to hospitalization  9. indication kidney replacement therapies  10. diuresis present  11. Dehydration  12. Any nephrotoxic Drug  13. leukocyte  14. transaminases levels  15. creatinine  16. use of vasopressors  17. mechanical ventilation | 0.77 | 0.78 | 0.58 | 0.89 | 0.82 |

*metrics performed in the validation cohort.

**Supplementary Table 03. Comparison of accuracy metrics in different models**

| Model | Predictors | INITIAL Avaible Predictors | Final Selected predictors | Accuracy | Sensitivity | Specificity | | Positive  predictive value | Negative  predictive value | ROC AUC* |
| --- | --- | --- | --- | --- | --- | --- | --- | --- | --- | --- |
| AKI-COV SCORE  (CURRENT Model) | Using feature selection^+^;  Excluded missing values more than 30% | 18 | 17 | 0.78 | 0.77 | | 0.78 | 0.58 | 0.89 | 0.82 [0.761-0.885] |
| LAsso model without feature selection | Without use of feature selection; excluded missing values more than 30% | 36 | 34 | 0.76 | 0.73 | | 0.77 | 0.58 | 0.87 | 0.81 [0.748-0.881] |
| LAsso model including all missing data | Without use of feature selection, using missing values more than 30% | 44 | 33 | 0.75 | 0.77 | | 0.76 | 0.53 | 0.90 | 0.81 [0.749-0.883] |

*metrics performed in the validation cohort.

+feature selection using Boruta algorithm

**Detailed steps to validate the AKI-COV in an independent data set:**

**1. Provide an independent data set with the same predictors used in the final model:** Age, Condition at Admission; AKI etiology; Hypertension; Sex; AKI source; Time COVID to AKI; Time COVID to hospitalization; indication kidney replacement therapies; diuresis present; Dehydration; Any nephrotoxic Drug; leukocyte; transaminases levels; creatinine; use of vasopressors

**2. Using the model to predict the outcome (class probability and classification).**

This step can be done fitting the final model with R code (provided as supplementary material) or using the calculator available online.

**3. Create a confusion Matrix and AUC-ROC** to compute the performance of the new data and compare it with the available model.
