# Supplementary material for: Development of a prediction score for in-hospital mortality in COVID-19 patients with acute kidney injury: a machine learning approach
Source: Sci Rep. 2021 Dec 24;11:24439. doi: 10.1038/s41598-021-03894-5 (PMC8709848; doi:10.1038/s41598-021-03894-5)
Supplement: Supplementary file 5 — Supplementary Information 5. [file 41598_2021_3894_MOESM5_ESM.pptx]

## Slide 1
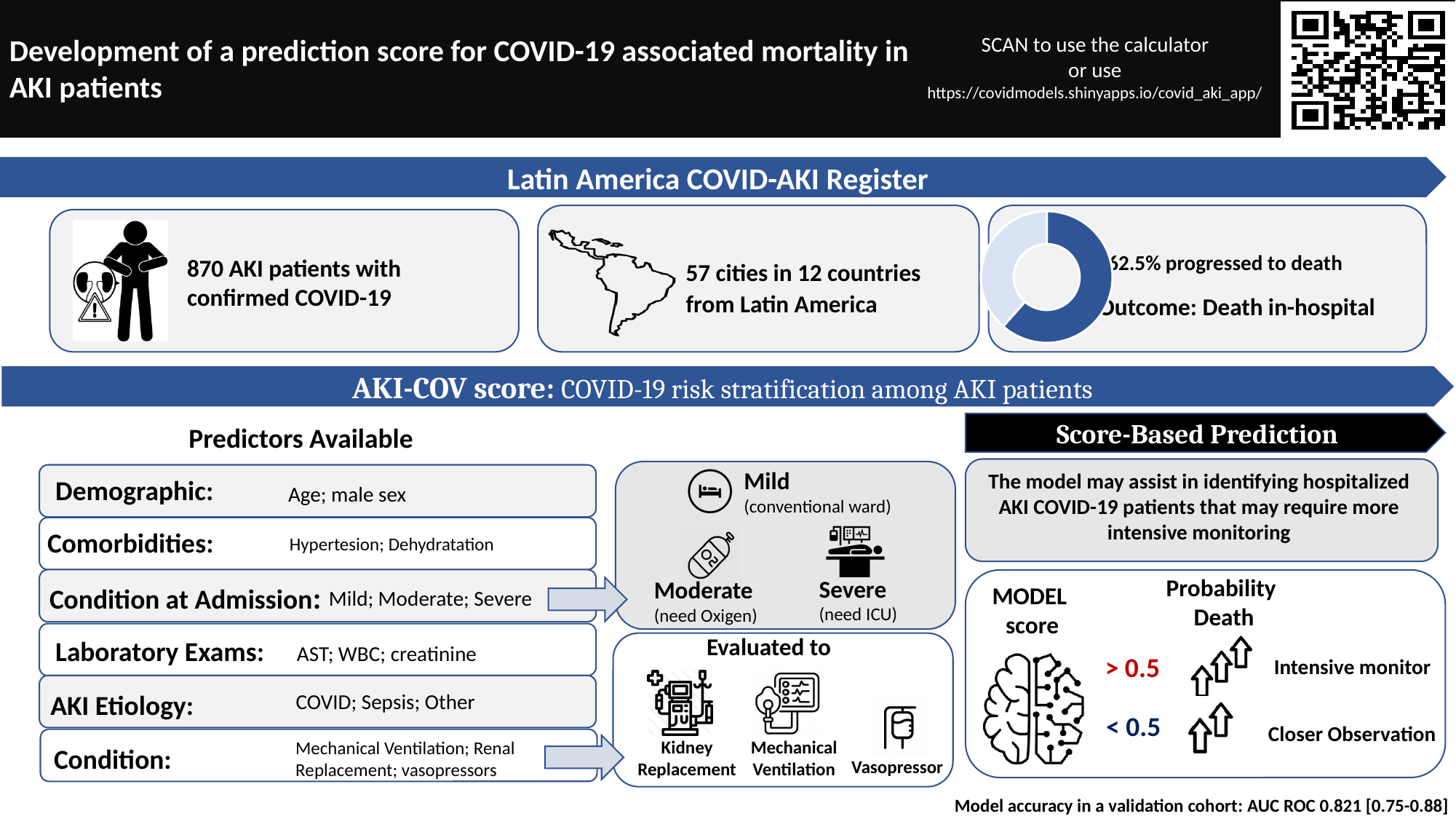

SCAN to use the calculator
or use
https://covidmodels.shinyapps.io/covid_aki_app/
Development of a prediction score for COVID-19 associated mortality in AKI patients
Latin America COVID-AKI Register
### Chart
| Category | |
|---|---|
62.5% progressed to death
870 AKI patients with confirmed COVID-19
57 cities in 12 countries from Latin America
Outcome: Death in-hospital
AKI-COV score: COVID-19 risk stratification among AKI patients
Score-Based Prediction
Predictors Available
Mild
(conventional ward)
The model may assist in identifying hospitalized AKI COVID-19 patients that may require more intensive monitoring
Demographic:
Age; male sex
Comorbidities:
Hypertesion; Dehydratation
Probability
 Death
Severe
(need ICU)
Moderate
(need Oxigen)
Condition at Admission:
MODEL
 score
Mild; Moderate; Severe
S
Evaluated to
Laboratory Exams:
AST; WBC; creatinine
> 0.5
Intensive monitor
AKI Etiology:
COVID; Sepsis; Other
< 0.5
Closer Observation
Mechanical
Ventilation
Kidney Replacement
Mechanical Ventilation; Renal Replacement; vasopressors
Condition:
Vasopressor
Model accuracy in a validation cohort: AUC ROC 0.821 [0.75-0.88]
